# Real or fake? Measuring the impact of protein annotation errors on estimates of domain gain and loss events

**DOI:** 10.3389/fbinf.2023.1178926

**Published:** 2023-04-20

**Authors:** Arnaud Kress, Olivier Poch, Odile Lecompte, Julie D. Thompson

**Affiliations:** Complex Systems and Translational Bioinformatics (CSTB), ICube laboratory UMR7357, University of Strasbourg, Strasbourg, France

**Keywords:** molecular evolution, protein domain analysis, protein analysis tools, sequence analysis, annotation error detection, primates, fungi

## Abstract

Protein annotation errors can have significant consequences in a wide range of fields, ranging from protein structure and function prediction to biomedical research, drug discovery, and biotechnology. By comparing the domains of different proteins, scientists can identify common domains, classify proteins based on their domain architecture, and highlight proteins that have evolved differently in one or more species or clades. However, genome-wide identification of different protein domain architectures involves a complex error-prone pipeline that includes genome sequencing, prediction of gene exon/intron structures, and inference of protein sequences and domain annotations. Here we developed an automated fact-checking approach to distinguish true domain loss/gain events from false events caused by errors that occur during the annotation process. Using genome-wide ortholog sets and taking advantage of the high-quality human and *Saccharomyces cerevisiae* genome annotations, we analyzed the domain gain and loss events in the predicted proteomes of 9 non-human primates (NHP) and 20 non-*S. cerevisiae* fungi (NSF) as annotated in the Uniprot and Interpro databases. Our approach allowed us to quantify the impact of errors on estimates of protein domain gains and losses, and we show that domain losses are over-estimated ten-fold and three-fold in the NHP and NSF proteins respectively. This is in line with previous studies of gene-level losses, where issues with genome sequencing or gene annotation led to genes being falsely inferred as absent. In addition, we show that insistent protein domain annotations are a major factor contributing to the false events. For the first time, to our knowledge, we show that domain gains are also over-estimated by three-fold and two-fold respectively in NHP and NSF proteins. Based on our more accurate estimates, we infer that true domain losses and gains in NHP with respect to humans are observed at similar rates, while domain gains in the more divergent NSF are observed twice as frequently as domain losses with respect to *S. cerevisiae*. This study highlights the need to critically examine the scientific validity of protein annotations, and represents a significant step toward scalable computational fact-checking methods that may 1 day mitigate the propagation of wrong information in protein databases.

## 1 Introduction

Extensive amounts of data from next-generation sequencing have led to the accumulation of information that provides insight into the evolutionary landscape of related proteins (Cheng et al., 2018; Lewin et al., 2018; Wu B. et al., 2022). Comparative analysis of these sequences has been pivotal to unravel mechanisms shaping evolution like gene duplication, loss and acquisition, and helps to shed light on genotype-phenotype associations (Dornburg et al., 2022; Schikora-Tamarit and Gabaldón, 2022). Comparative analysis relies on the identification of sets of orthologous and paralogous genes and subsequent transfer of structural and functional annotations (Stamboulian et al., 2020). However, the advances of comparative genomics have made it clear that the exclusive focus on genes as units of evolution is an over-simplification of the actual evolutionary relationships (Jain et al., 2019; Yu et al., 2019; Nevers et al., 2020). Gene-level methods are limited especially when comparing complex multi-domain proteins or phylogenetically distant species. To overcome these bottlenecks, protein domains have been suggested as an alternative unit for studying groups of functionally equivalent proteins (Gabaldon and Koonin, 2013; Syamaladevi et al., 2013; Wang et al., 2021). Differences in protein domain composition can be common among orthologs and are functionally important, especially for multicellular eukaryotes where domain architectures are often highly complex and lineage specific (Sjolander et al., 2011; Forslund and Sonnhammer, 2012; Persson et al., 2019). Underscoring their importance, ortholog databases are beginning to include domain level information (Forslund et al., 2018; Nevers et al., 2022).

The most common changes observed in both ortholog and paralog pairs involved loss/gain of domains, while domain shuffling and segment duplication/deletion are less frequent (Forslund et al., 2011). However, there has been some debate about the true level of domain conservation of orthologs (Lin et al., 2006; Forslund et al., 2011; Nagy et al., 2011; Dohmen et al., 2020). Notably, a comparison of domain architectures for orthologous proteins in the high quality manually curated UniProtKB/Swiss-Prot database (Uniprot Consortium, 2022) suggested that the rate of architecture alteration is very low: the domain architecture of ∼5% of the orthologs is changed over 1,000 My (Nagy et al., 2011). In contrast, comparison of UniProtKB/TrEMBL or RefSeq predicted protein sequences identified a significantly higher proportion of differences, for example 4.01% of human-mouse orthologs in TrEMBL had different architectures compared to 1.1% in SwissProt. The explanation for this difference is that the predicted sequence databases are significantly contaminated with incomplete or chimeric protein sequences that have missing domain annotations compared to their complete Swiss-Prot orthologs. Since then, a growing body of evidence has revealed significant levels of other types of protein annotation errors in public databases (Schnoes et al., 2009; Steinegger and Salzberg, 2020; Rembeza and Engqvist, 2021; Goudey et al., 2022).

The starting point for the complex process of protein domain annotation is the availability of high quality genomes. For technical and methodological reasons, available genome sequences are of different quality: some are complete, while others are incomplete or in draft state (http://www.genomesonline.org/cgi-bin/GOLD/index.cgi). The subsequent genome annotations of gene intron/exon structures are also of highly diverse quality. For model genomes, for example from human, mouse or the yeast *Saccharomyces cerevisiae*, extensive transcriptome data is available allowing more accurate gene identification (Weirather et al., 2017). For other organisms, the genomes are automatically annotated and gene/protein sequences have not been fully verified. This is particularly serious for draft genomes, misleading on the actual number of genes/isoforms (Deutekom et al., 2019; Weisman et al., 2022) and the corresponding protein sequences (Bányai and Patthy, 2016; Tørresen et al., 2019; Meyer et al., 2020). Finally, it has been shown that default protein domain annotations, generally based on searches for known protein domains listed in public databases like Interpro (Blum et al., 2021) or Pfam (Mistry et al., 2021), can be inconsistent between species (Nagy et al., 2011). For example, the use of stringent parameters means that a Pfam-A domain identified in ortholog A might be undetected in ortholog B at the same cut-off value. This type of error was observed to occur notably in the case of small domains or less conserved domains where E-values tend to be close to the selected cut-off value.

This article investigates the effects of systematic errors on the ability to identify true domain events. We developed an automated fact-checking method that identifies errors at three main levels: genome sequencing/assembly, gene prediction, and domain identification. The protocol relies on the availability of high quality protein sequences from a model species, which we call the reference species, and takes advantage of the single copy (or one-to-one) orthologs widely used for evolutionary studies and functional inferences (Nevers et al., 2020). We apply the method to two genome-scale ortholog sets containing the predicted proteomes of 9 non-human primates (NHP) and 23 non-*S. cerevisiae* fungi (NSF) respectively from the UniprotKB database, and evaluate all potential domain gain and loss events using the well-studied human and *S. cerevisiae* proteomes as a reference. However, the method should be easily applicable to other ortholog sets, where it can be used to filter the potential domain events that are due to annotation errors, thus highlighting candidates for true evolutionary novelty.

## 2 Materials and methods

### 2.1 Protein sequence data

Human and *S. cerevisiae* proteins were identified in the Uniprot database version 2022_03. For each protein, one-to-one orthologs for 9 NHP and 21 NSF species were then extracted from the OrthoInspector database (v3) (Nevers et al., 2019). For all proteins, the canonical sequences were retrieved from Uniprot and domain architectures were retrieved from the Interpro database (Pfam-A only) using the API in json format. Protein sequences longer than 5,000 amino acids were eliminated for convenience. The corresponding genomic sequences were then retrieved from the Ensembl database (Martin et al., 2022), using the pre-defined gene positions extended by + -1,000 nucleotides. Genome level information was also obtained from Ensembl, including the total number of coding genes and N50 statistics. The contig N50 is defined as the length of the shortest contig for which longer and equal length contigs cover at least 50% of the assembly.

For all genomes, we also collected BUSCO complete metrics as provided in (Manni et al., 2021). BUSCO is a complementary measure used to assess genome assembly and gene annotation completeness. It reflects the percentage of complete and fragmented genes relative to a Benchmark of universal Single-Copy Orthologs.

### 2.2 Identification of different domain contents

**FIGURE 1:**
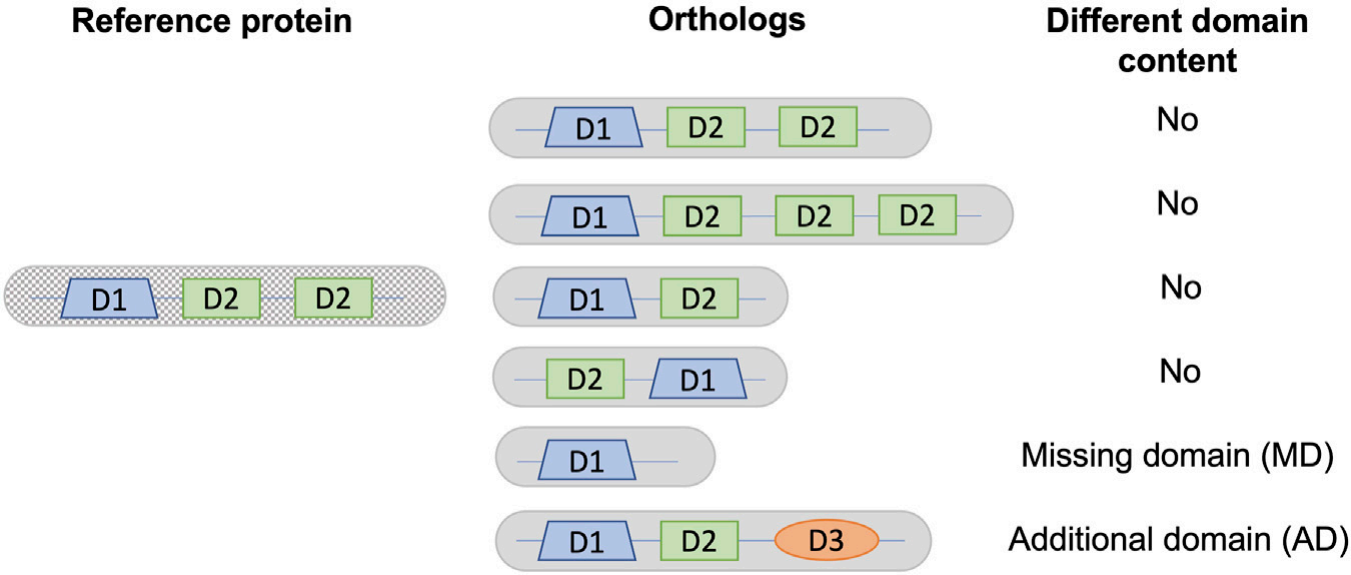
Definition of different domain content used in this analysis. As an example, a reference protein is shown with a single copy of domain D1 and two copies of domain D2. Different domain content is defined as a change in the list of distinct domains, corresponding to either a missing domain (MD) or an additional domain (AD) in a given ortholog from a non-reference species with respect to the reference protein.

We compared the Pfam-A domain annotations of each orthologous protein with the human or *S. cerevisiae* reference protein as appropriate, identifying sequences that had either missing or additional domains. The domain content of a protein was defined as the list of distinct Pfam-A domains. Only distinct domains were taken into account, ignoring duplication events that create new copies of the same domain. Architectures with either different domain counts or order were considered to have the same domain content (Figure 1). Domains that overlapped over at least half of their lengths in the reference sequence were grouped into a single entry. Thus, if one of the overlapping domains was present in an orthologous sequence, the domain content was considered to be the same. For sequences with repeated domains, the domain content was considered to be the same if an orthologous sequence contained at least one instance of the repeated domain.

### 2.3 Identification of inconsistent domain annotations

For all ortholog pairs with different domain contents, the domain annotations were re-computed using the same protocol. Pfam HMM-profiles (version 35.0 November 2021) were downloaded from http://ftp.ebi.ac.uk/pub/databases/Pfam/current_release/Pfam-A.hmm.gz and installed locally. Then, HMMSEARCH (version 3.3.2) was used to search each protein sequence against the Pfam HMM-profiles with an E-value threshold of 0.01 and the gathering (GA) threshold option.

### 2.4 Identification of potential protein sequence prediction errors

For each orthologous sequence with a different domain composition, the prosplign algorithm implemented in the NCBI Genome Workbench (Kuznetsov and Bollin, 2021) was used to build a pairwise alignment between the corresponding gene sequence and the human/*S. cerevisiae* protein sequence. A new predicted protein sequence was then extracted from the prosplign alignment using an in-house script. To evaluate the new predicted sequences, we searched the Pfam HMM-profiles (version 35.0) against the sequences using the HMMER tool (version 3.3.2) with E-value threshold of 0.01 and the gathering (GA) threshold option.

### 2.5 Identification of potential genome sequence errors

The genomic sequence coding for the ortholog proteins (NHP/NSF) were parsed to identify undetermined nucleotides, represented by ‘N’ characters.

### 2.6 Ordinal position analysis

To investigate whether missing domain (MD) or additional domain (AD) events occurred predominantly at the N/C-termini of proteins rather than internally, we determined the ordinal positions of true MD/AD events with respect to the domain architecture. To do this, we considered only ortholog pairs where both proteins had at least two domains. For each protein, the annotated domains were ordered from the N-terminus to the C-terminus, and the position of the MD/AD was classed as either N-terminal, C-terminal or internal.

### 2.7 Classification of Pfam-A domains into clans

The domain entries in Pfam are grouped hierarchically into clans if they are considered to have arisen from a single evolutionary origin, assessed by the presence of related 3D structures, related functions, significant matching of the same sequence to HMMs from different families and profile–profile comparisons. We retrieved the clan classification for all Pfam-A domain entries from the Interpro resource at https://ftp.ebi.ac.uk/pub/databases/Pfam/current_release/ Pfam-A.clans.tsv.gz.

### 2.8 Phylogenetically corrected regression analysis (PGLS)

To estimate the phylogenetic distance between the reference species (human/*S. cerevisiae*) and non-reference species (NHP/NSF), (i) for NHP, we downloaded the species tree from the Ensembl database, and (ii) for NSF, we retrieved a recently published genome-level phylogeny (Li et al., 2021). The trees were then used to calculate patristic distances (i.e. sum of branch lengths) between species. Finally, to test for correlations between the number of domain events observed and the phylogenetic distance, we used the phylogenetic generalized least squares (PGLS) method implemented in the CAPER (http://cran.r-project.org/package=caper) v0.5 package for R. Following standard practice, delta (δ) and kappa (κ) were set to 1 while the maximum-likelihood value of lambda (λ) was estimated and used to transform branch lengths.

## 3 Results

### 3.1 Protocol for fact checking the domain annotation process

We developed an automated method to verify each stage of the annotation process and to distinguish true MD/AD events from false events caused by protein annotation errors. The protocol takes as input a set of high quality protein sequences from a reference species, and their one-to-one orthologs from the selected non-reference species. In contrast to the reference species, many of the proteins for the non-reference species are predicted and have not been confirmed experimentally.

For all proteins, we defined their domain content as the list of distinct domains predicted to be present in the protein. We then identified potential domain events by comparing the domain contents for a given ortholog pair, consisting of a reference sequence and a non-reference ortholog sequence. We focused on domain events involving loss or gain of new domains, rather than duplications or rearrangements of existing domains leading to a different order or number of domains, as shown in Figure 1.

**FIGURE 2:**
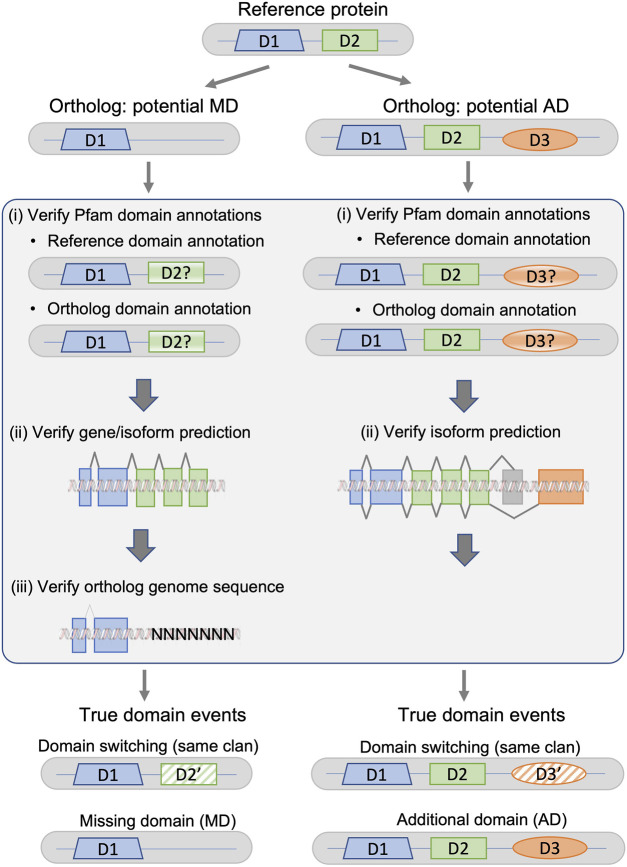
Fact checking protocol for potential missing domain (MD) and additional domain (AD) events in a given ortholog from a non-reference species with respect to the protein from the reference species. True domain events are classified as either domain switching (the ortholog sequence does not contain domain D2, but contains a domain D2′ belonging to the same Pfam clan as D2), or a MD/AD event.

The protocol, summarized in Figure 2, is designed to detect different types of systematic failures, including genome sequencing/assembly, gene or isoform prediction, and protein domain annotation.

### 3.1.1 Verification of annotated protein domains

For both MD and AD, the first step is the identification of inconsistent protein domain annotations. The domain annotations for all reference and non-reference proteins were extracted from the Interpro database, using only Pfam-A domains. The annotations in the Pfam database are constructed using a combination of human expert curation and automatic predictions from statistical models. InterPro also integrates predictive models from several other databases, and does not apply a single E-value threshold applied to all entries. This generally improves accuracy, but can lead to inconsistent annotations between organisms (). To identify inconsistent domain annotations, we re-annotated the proteins that had different domain contents using a standardized protocol based on hmmsearch queries against a locally installed version of the PFAM-HMM domain profiles, and a defined E-value threshold of 0.01.

### 3.1.2 Verification of gene/isoform predictions

For potential MD events in the non-reference orthologs, the second step involves verification of the gene/isoform prediction and the inferred protein sequence. We extracted the genomic sequence coding for the non-reference protein from the Ensembl database, extending the gene sequence 1,000 nucleotides upstream and downstream of the defined gene region. We then used TBLASTN to search for a genome sequence region potentially coding for the missing domain. For potential AD events in the ortholog proteins, the gene prediction verification was unnecessary, since the domain is present in the non-reference ortholog, but absent from the reference protein. Gene predictions in these well-studied genomes were considered to be correct. However, it is possible that there are inconsistencies in the definition of the canonical isoforms in Uniprot, and that the canonical sequence defined in the non-reference organism is orthologous to an alternatively spliced reference protein. Therefore, the second step involves searching for known alternative isoforms of the reference protein that contain the missing domain. For each reference protein, alternative isoforms were retrieved from the Ensembl database, which includes data from UniProtKB/Swiss-Prot, UniProtKB/TrEMBL, and RefSeq.

### 3.1.3 Verification of genome sequence/assembly

The third step for the potential MD events involves verification of the original genome sequence for the non-reference species. Specifically, we searched for uncharacterized regions that are indicated by ‘N’ characters in the sequence. Again, this step is unnecessary in the case of AD events, since the domain is absent from the reference protein and the reference genome is considered to be correct.

If no errors or inconsistencies were found in any of the error detection steps, the potential MD/AD events were considered to be true.

### 3.2 Construction of genome-scale data sets

We applied our protocol to two data sets containing: (i) 9 NHP proteomes (Table 1) which were compared to the reference human proteome, (ii) 20 NSF proteomes (Table 2) compared to the reference *S. cerevisiae* proteome. All protein sequences were extracted from the UniprotKB database. The two datasets represent some of the main challenges for domain annotation pipelines. First, the NHP species are closely related (with proteomes sharing >89% sequence identity with human), but proteins are generally more complex, with multi-domain architectures. Furthermore, some of the genome assemblies are incomplete, as estimated by their contig N50 metric of <100 Kb compared to >10 Mb for the higher quality genomes or 55 Mb for the human genome. The contig N50 is defined as the length of the shortest contig for which longer and equal length contigs cover at least 50% of the assembly. The NSF represent more divergent species (mean sequence identity ranging from 24.5% to 41.8% with *S. cerevisiae*). However, the genomes are smaller and gene prediction is simpler since the genes are less intron-rich.

**TABLE 1:** Non-human primates (NHP) species used in the analysis. BUSCO comp_V5 refer to BUSCO completeness metrics from (Manni et al., 2021).

Primates	Reference genome	Uniprot code	TAXID	Contig N50	BUSCO comp_V5	No. of coding genes	No. of orthologs	Mean % identity
Apes								
Chimpanzee (*Pan troglodytes*)	GCA_000001515.5	PANTR	9598	384,816	96.0	23,534	17,944	98.5
Gorilla (*Gorilla gorilla*)	GCA_000151905.3	GORGO	9595	52,934	97.2	21,794	17,670	98.2
Orangutan (*Pongo abelii*)	GCA_002880775.3	PONAB	9601	11074009	99.3	20,211	16,490	97.3
Gibbon (*Nomascus leucogenys*)	GCA_000146795.3	NOMLE	61,853	35,148	99.7	20,794	16,797	96.5
Old world monkeys								
Baboon (*Papio anubis*)		PAPAN	9555	1465768	98.6	21,882	17,107	96.1
Vervet (*Chlorocebus sabaeus*)	GCA_000409795.2	CHLSB	60,711	90,449	99.0	19,165	17,192	96.2
Macaque (*Macaca mulatta*)	GCA_003339765.3	MACMU	9544	46608966	98.8	21,761	16,255	95.9
New world monkeys								
Marmoset (*Callithrix jacchus*)	GCA_011100555.1	CALJA	9483	13222669	99.9	22,078	16,714	94.0
Prosimians								
Bushbaby (*Otolemur garnettii)*	(GCA_000181295.3	OTOGA	30,611	27,100	97.4	19,506	16,071	89.4
Total							152,240	

**TABLE 2:** Non- *Saccharomyces cerevisiae* fungi (NSF) species used in the analysis.

Fungi	Reference genome	Uniprot code	TAXID	Contig N50	BUSCO comp_V5	No. of coding genes	No. of orthologs	Mean % identity
Ascomycota								
*Yarrowia lipolytica*	GCA_000002525.1	YARLI	284,591	182,835	95.7	6,448	2,889	36.1
*Schizosaccharomyces pombe*	GCA_000002945.2	SCHPO	284,812	2923134	79.9	5,145	2,340	32.9
*Arthrobotrys oligospora*	GCA_000225545.1	ARTOA	756,982	5390931	95.4	11,479	2,641	32.1
*Tuber melanosporum*	GCA_000151645.1	TUBMM	656,061	63,046	92.8	7496	2,537	31.9
*Aspergillus fumigatus*	GCA_002234955.1	ASPFU	330,879	556,577	96.6	9623	2,561	31.7
*Neurospora crassa*	GCA_000182925.2	NEUCR	367,110	102,005	97.9	9758	2,615	31.5
*Eutypa lata*	GCA_000349385.1	EUTLA	1287681	190,808	97	11,685	2,350	31.0
*Phaeosphaeria nodorum*	GCA_000146915.1	PHANO	321,614	16,751	93.2	12,391	2,502	30.3
Basidiomycota								
*Cryptococcus neoformans*	GCA_000091045.1	CRYNJ	214,684	1423448	98.7	6,632	2,177	29.7
*Coprinopsis cinerea*	GCA_000182895.1	COPC7	240,176	3468139	98.8	13,355	2,161	29.6
*Wallemia ichthyophaga*	GCA_004918415.1	WALI9	1299,270	436,658	87	4,865	1997	29.5
*Ustilago maydis*	GCA_000328475.2	USTMA	237,631	111,545	99.1	6,765	2,199	29.0
*Microbotryum violaceum*	GCA_000166175.1	USTV1	683,840	1329596	94.4	7364	2,202	28.8
*Puccinia graminis*	GCA_000149925.1	PUCGT	418,459	53,646	88.8	15,800	1849	28.5
*Mixia osmundae*	GCA_000708205.1	MIXOS	764,103	426,173	88.4	6,726	2,111	28.1
Blastocladiomycota								
*Allomyces macrogynus*	GCA_000151295.1	ALLMA	578,462	35,497	83.9	18,774	402	28.2
Chytridiomycota								
*Spizellomyces punctatus*	GCA_000182565.2	SPIPN	645,134	155,888	90.9	8950	2,100	29.8
*Gonapodya prolifera*	GCA_001574975.1	GONPJ	1344416	63,757	74.8	13,827	1819	29.1
Microsporidia								
*Nosema ceranae*	GCA_000988165.1	NOSCE	578,460	42,592	93.5	2060	467	25.2
*Vavraia culicis*	GCA_000192795.1	VAVCU	948,595	94,471	95.5	2,773	491	24.5
Total							40,410	

We then identified all ortholog pairs between each reference proteome (human, *S. cerevisiae*) and the corresponding non-reference proteomes (NHP, NSF respectively). Orthologs were extracted from the Ortholnspector database and the sequences that were cross-referenced in the Ensembl genome database were retained. In order to avoid redundancy and bias in the data sets, only one-to-one orthologs were considered. For the primate dataset, this resulted in 19,251 human proteins (out of 19,813) that had at least one-to-one ortholog, and a total of 152,240 orthologous NHP sequences (Table 1). Of the 152,240 orthologous sequences, 2,264 (1.5%) were extracted from the SwissProt database, and the remaining 149,976 (98.5%) were from TrEMBL. The number of orthologous NHP sequences retained is generally similar for each species, ranging from 16,071 to 17,944. For the fungi dataset, 3404 *S. cerevisiae* proteins (out of 6,600) were retained and a total of 40,410 orthologous NSF sequences (Table 2). Of the 40,410 orthologous sequences, 4,853 (13.6%) were extracted from SwissProt, and 355,557 (86.4%) were from TrEMBL. The proportion of *S. cerevisiae* proteins with one-to-one orthologs is smaller (52%) compared to human proteins (97%), due to the higher divergence of the selected species in the fungi dataset. The number of orthologous NSF sequences retained for each species ranges from 1819–2,889, with the exception of the two obligate unicellular parasites (*Nosema ceranae* and *V. culicis*) that share less than 500 orthologs with *S. cerevisiae,* and *A. macrogynus* that has a higher level of gene duplications resulting in a larger proportion of one-to-many orthologs and only 402 one-to-one orthologs.

### 3.3 Identification of all potential MD and AD events

For all proteins, we retrieved their Pfam-A domain annotations from the Interpro database and compared the domain contents of all ortholog pairs, where an ortholog pair consists of a protein from the reference species and its one-to-one ortholog in a non-reference species.

For the primate dataset (Table 3), a total of 5,695 (3.6%) of the 152,240 orthologous NHP sequences had different domain contents, with 4,462 orthologs presenting a potential missing domain (MD) with respect to human and 1,233 orthologs presenting a potential additional domain (AD). Interestingly, only 2 (0.04%) of the 4462 MD and 4 (0.3%) of the 1233 AD were found in sequences from the SwissProt database. The number of potential MD varied from 237 for chimpanzee to more than 700 for gibbon and vervet*.* The number of potential AD was smaller for all NHP species, ranging from 92 to 199, and leading to a mean MD/AD ratio of 3.6. The MD/AD ratio varied significantly from 1.9 for macaque to 6.1 for vervet.

**TABLE 3:** Potential domain events identified in NHP orthologs with respect to human proteins.

Primate	Potential MD	Potential AD	Ratio MD/AD	Total	% orthologs with domain events
Chimpanzee	237	92	2.6	329	1.8
Gorilla	480	102	4.7	582	3.3
Orangutan	400	121	3.3	521	3.1
Gibbon	763	131	5.8	894	5.3
Baboon	422	154	2.7	576	3.3
Vervet	746	122	6.1	868	5.0
Macaque	388	199	1.9	587	3.6
Marmoset	349	139	2.5	488	2.9
Bushbaby	677	173	3.9	850	5.2
Total	4,462	1,233	3.6	5,695	3.7

For the fungi dataset (Table 4), a total of 7772 of the 40,410 orthologous sequences had different domain contents, i.e. 17.6%, with 3987 orthologs (including 233 or 6.2% from SwissProt, and 3754 or 93.8% from TrEMBL) presenting a MD with respect to *S. cerevisiae* and 3785 orthologs (including 289 or 7.6% from SwissProt, and 3496 or 92.4% from TrEMBL) presenting an AD. The Microsporidia parasites *N. ceranae* and *V. culicis* had a smaller number of potential AD (15 and 14 respectively) as might be expected since they are obligate unicellular parasites with a smaller proteome (467 and 491 protein orthologs respectively), but a much larger number of MD (178 and 191 respectively). For the remaining NSF, the MD/AD ratio varied from 0.5 (*i.e.* AD are twice as frequent as MD) for *Spizellomyces punctatus* to 1.6 (*i.e.* MD are more frequent than AD) for *E. lata* and *A. macrogynus*. No correlation was observed between the MD/AD ratios and the NSF clades, but the overall frequency of events per ortholog was generally lower for the Ascomycota, with on average 14% of orthologs with domain events, compared to Basidiomycota, Blastocladiomycota and Chytridiomycota, with 19%, and Microsporidia with 41%.

**TABLE 4:** Potential domain events identified in NSF orthologs with respect to *Saccharomyces cerevisiae* proteins.

Fungi	Potential MD	Potential AD	Ratio MD/AD	Total	% orthologs with domain events
*Yarrowia lipolytica*	186	163	1.1	349	11.7
*Schizosaccharomyces pombe*	130	174	0.7	304	12.4
*Arthrobotrys oligospora*	193	207	0.9	400	14.8
*Tuber melanosporum*	236	192	1.2	428	16.4
*Aspergillus fumigatus*	164	199	0.8	363	13.4
*Neurospora crassa*	156	195	0.8	351	13.1
*Eutypa lata*	294	183	1.6	477	19.5
*Phaeosphaeria nodorum*	245	237	1.0	482	18.6
*Cryptococcus neoformans*	181	174	1.0	355	15.7
*Coprinopsis cinerea*	210	212	1.0	422	18.4
*Wallemia ichthyophaga*	211	277	0.8	488	23.9
*Ustilago maydis*	180	221	0.8	401	17.7
*Microbotryum violaceum*	195	211	0.9	406	17.8
*Puccinia graminis*	232	159	1.5	391	18.5
*Mixia osmundae*	181	316	0.6	497	22.5
*Allomyces macrogynus*	273	173	1.6	446	21.0
*Spizellomyces punctatus*	144	302	0.5	446	19.8
*Gonapodya prolifera*	207	161	1.3	368	18.1
*Nosema ceranae*	178	15	11.9	193	40.6
*Vavraia culicis*	191	14	13.6	205	40.9
Total	3987	3785	1.1	7772	17.6

### 3.4 Verification of potential missing domain (MD) events

We used our fact checking approach (Figure 2) to evaluate all non-reference (NHP/NSF) proteins that had potential MD events according to the Pfam-A domain annotations in the Interpro database.

First, for the 4462 NHP orthologs and 3987 NSF orthologs with potential MD compared to the reference (human/*S. cerevisiae*), we identified 1713 (38%) and 2008 (50%) MD events for NHP and NSF respectively linked to inconsistent domain annotations. These false positive MD events include 1,147 and 1,614 cases for NHP and NSF respectively, where the MD was identified in the orthologous sequences by our protocol. In other words, while the NHP/NSF protein was not annotated with the domain in the Interpro database, increasing the hmmsearch E-value allowed us to identify the MD. As an illustrative example, the human protein P55318 (FOXA3_HUMAN: transcription factor forkhead box protein 3) and the macaque ortholog F6Q3J5 both contain a central forkhead domain (PF00250), but the forkhead N-terminal region (PF08430) is not annotated in the macaque sequence. The N-terminal region was found in the human sequence with an hmmsearch E-value = 3.9 × 10^−10^, and in the macaque sequence with E-value = 1.6 × 10^−8^. This domain is also identified by a new Deep Learning method (Bileschi et al., 2022) that was trained on Pfam data and annotated as a Pfam-N (N for Network) domain in Interpro.

More surprisingly, we discovered 566 and 394 cases for NHP and NSF respectively, where the MD was not detected in the reference sequence by our protocol. Thus, the protein is annotated with the domain by Interpro, but is not detected by hmmsearch with an E-value threshold of 0.01. As an example, the *S. cerevisiae* protein Q05580 (HEL2_YEAST) is annotated with the Interpro domain “Zinc finger C2H2-type” (IPR013087), which combines signatures from two member databases SMART (SM00355) and Pfam (PF00096). The annotation file retrieved from Interpro contains both signatures, even though the Pfam signature does not match this specific protein with an E-value below the threshold.

**FIGURE 3:**
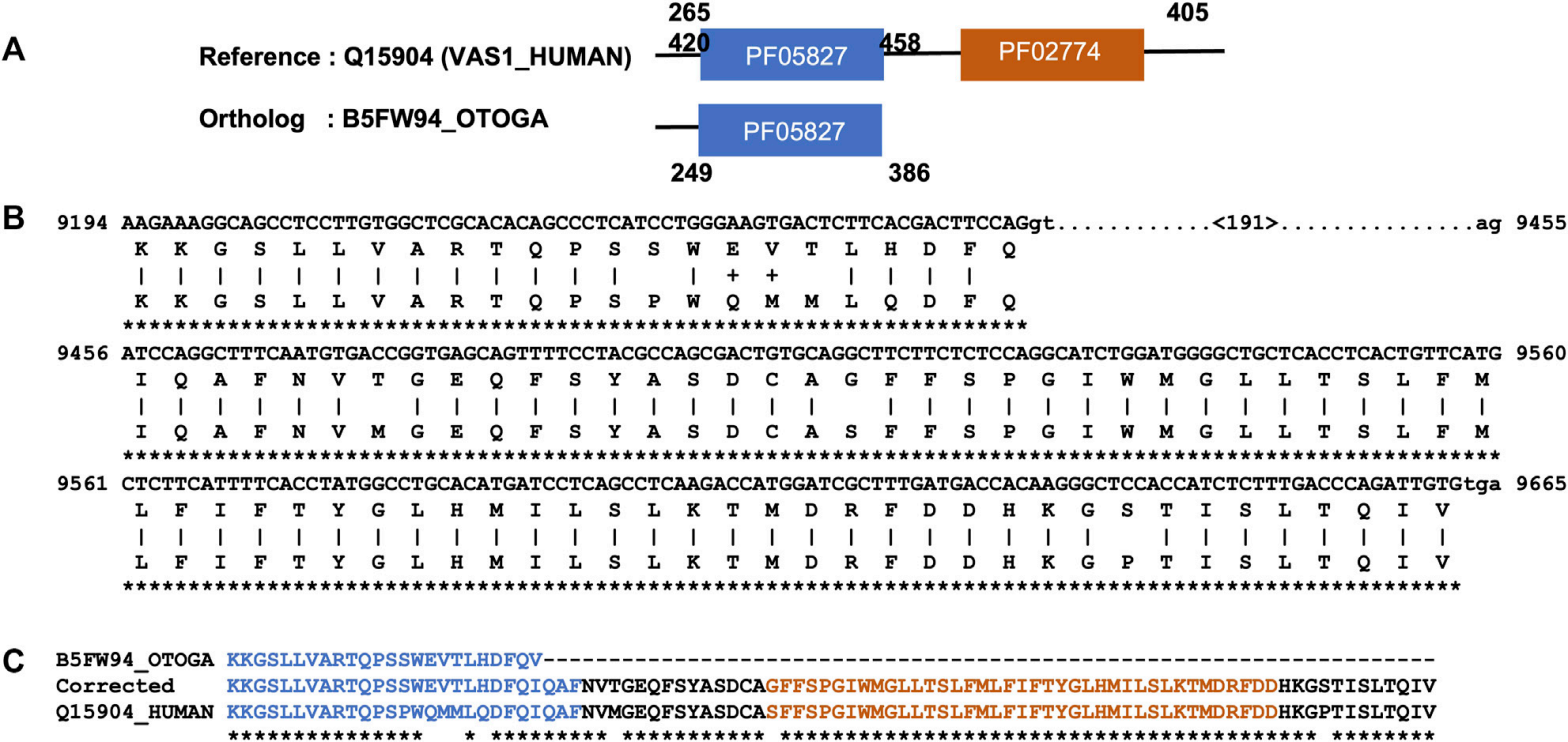
Example of a MD event due to a gene prediction error. **(A)**. Domain annotations extracted from the Interpro database, with a potential MD in the bushbaby sequence (B5FW94). **(B)**. Part of the Tblastn alignment identifying an additional exon at the 3′ end of the corresponding genome region (Ensembl: ENSOGAG00000008310). Exons are shown in uppercase and introns in lowercase. The number in brackets indicates the length of the potential intron. **(C)**. Part of the protein sequence alignment of the human sequence (Q15904), the B5FW94 bushbaby sequence, and the corrected bushbaby sequence with the C-terminal extension coded by the additional exon.

Next, we focused on the identification of gene/isoform prediction errors for the remaining 2749 NHP and 1979 NSF potential MD events that could not be explained by domain annotation issues. After applying a simple correction protocol based on alignment of the reference protein sequence with the genomic sequence of the ortholog (see Methods), the MD was identified in 1368 NHP and 464 NSF orthologs. An illustrative example is shown in Figure 3 for the potential MD event in the bushbaby sequence B5FW94, with respect to the human sequence Q15904 (VAS1_HUMAN). VAS1_HUMAN is described as an accessory subunit of the proton-transporting vacuolar (V)-ATPase protein pump, which is essential in supporting intracellular membrane trafficking and protein degradation, which in turn are important for immune responses, cell signaling, and neurotransmitter release (Wang et al., 2020). The human protein contains a luminal domain (PF05827), as well as a C-terminal transmembrane domain (PF02274) that is potentially lost in the bushbaby ortholog B5FW94. However, a search of the corresponding bushbaby genome identifies a sequence segment downstream of the gene that could represent an additional exon coding for the MD.

Finally, in step 3, we identified genome sequence issues, where 970 NHP domains and 272 NSF domains had a corresponding genome sequence containing undetermined characters (N’s). As a consequence, it is difficult to determine whether the missing domain is present or not.

**FIGURE 4:**
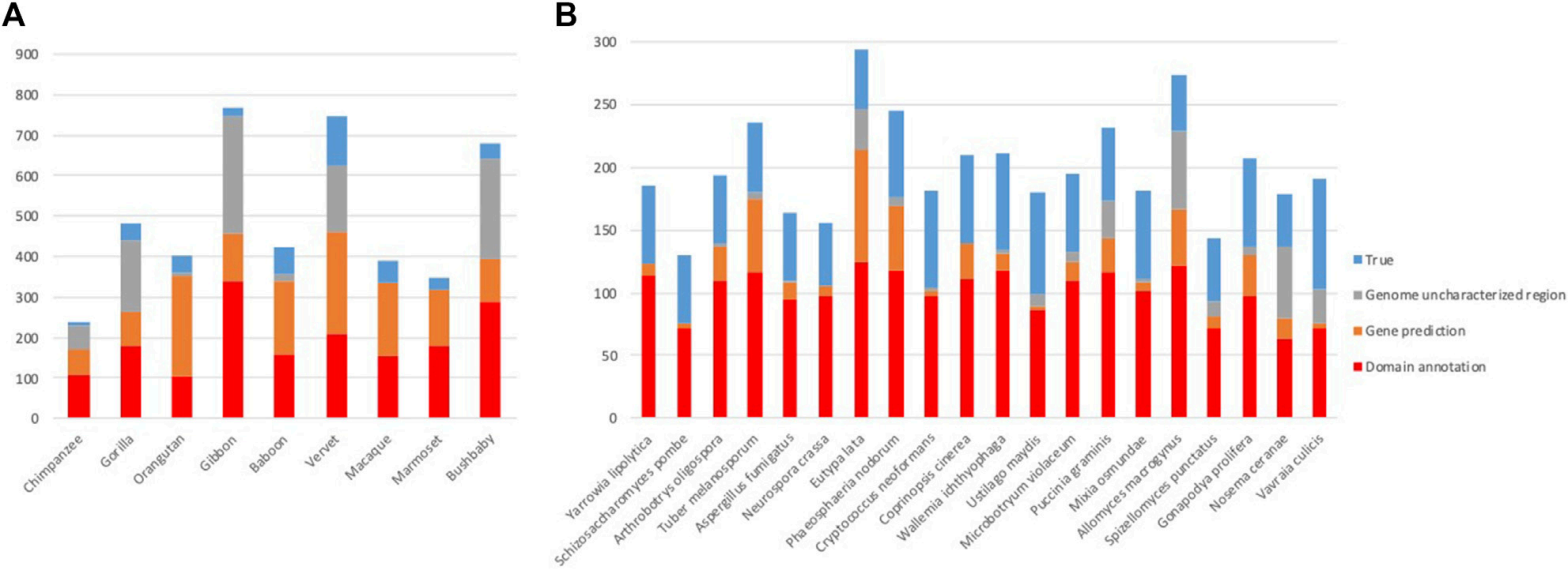
Results of error detection protocol for **(A)** NHP proteins and **(B)** NSF proteins with potential MD.

After the three stages of error detection (Figure 4; Table 5, Table 6), only 411 NHP MD events out of 4,462 (9.2%) and 1243 NSF MD events out of 3987 (31.2%) are considered to be candidates for true domain events. Of these, 0 NHP MD events and 99 (8.6%) NSF MD events were found in SwissProt proteins.

**TABLE 5:** Results of error detection protocol for NHP proteins with potential missing domain (MD) events. % true MD = % of potential MD that are identified as true MD after re-evaluation. Numbers in brackets represent the percentage of potential MD linked to the 3 different types of errors (domain annotation, gene/isoform prediction and undetermined genome region).

	Potential MD	Domain annotation	Gene/isoform prediction	Undetermined genome regions	True MD	% true MD
Chimpanzee	237	107	63	57	10	4.2
Gorilla	480	180	81	180	39	8.1
Orangutan	400	103	246	11	40	10.0
Gibbon	763	337	116	293	17	2.4
Baboon	422	160	181	15	66	15.6
Vervet	746	208	253	164	121	16.2
Macaque	388	155	182	0	51	13.1
Marmoset	349	178	139	2	30	8.6
Bushbaby	677	285	107	248	37	5.5
Total	4,462	1713	1,368	970	411	9.2

**TABLE 6:** Results of error detection protocol for NSF proteins with potential missing domain (MD) events.

	Potential MD	Domain annotation	Gene prediction	Undetermined genome regions	True MD	% true MD
*Yarrowia lipolytica*	186	114	9	0	63	33.9
*Schizosaccharomyces pombe*	130	72	3	0	55	42.3
*Arthrobotrys oligospora*	193	109	27	3	54	28.0
*Tuber melanosporum*	236	116	58	6	56	23.7
*Aspergillus fumigatus*	164	95	13	1	55	33.5
*Neurospora crassa*	156	97	9	0	50	32.1
*Eutypa lata*	294	124	90	33	47	16.0
*Phaeosphaeria nodorum*	245	118	51	7	69	28.2
*Cryptococcus neoformans*	181	97	4	2	78	43.1
*Coprinopsis cinerea*	210	111	28	1	70	33.3
*Wallemia ichthyophaga*	211	117	14	3	77	36.5
*Ustilago maydis*	180	86	3	9	82	45.6
*Microbotryum violaceum*	195	109	15	9	62	31.8
*Puccinia graminis*	232	116	27	30	59	25.4
*Mixia osmundae*	181	101	7	3	70	38.7
*Allomyces macrogynus*	273	122	44	63	44	16.1
*Spizellomyces punctatus*	144	72	9	12	51	35.4
*Gonapodya prolifera*	207	97	33	6	71	34.3
*Nosema ceranae*	178	63	17	56	42	23.6
*Vavraia culicis*	191	72	3	28	88	46.1
Total	3987	2008	464	272	1,243	31.2

### 3.5 Verification of potential additional domain (AD) events

The first step is the identification of inconsistent protein domain annotations, as described in the previous section for MD. For the 1233 NHP orthologs and 3785 NSF orthologs with potential AD events compared to the reference (human/*S. cerevisiae*), 716 and 1,622 cases for NHP and NSF respectively were identified as false positives due to inconsistent domain annotations. These include 183 and 382 cases where the potentially gained domain was not identified in the NHP and NSF ortholog sequence respectively, as well as 533 and 1,240 cases where the AD was found in the reference sequence (human/*S. cerevisiae*) by our protocol. As an example, protein H2P1R9_PONAB is an ortholog of the human sequence Q96LM9 (CT173_HUMAN), an uncharacterized protein with an annotation score of 2/5 in Uniprot. Both proteins are annotated in Interpro as belonging to the GT29-like superfamily (IPR038578), but CT173_HUMAN has no Pfam annotations. H2P1R9_PONAB is annotated with a glycosyltransferase family 29 domain (PF00777) in the Interpro database and the domain is also identified using hmmsearch with an E-value = 2.0e-10. Using hmmsearch, PF00777 is also found in the CT173_HUMAN sequence with E-value = 3.2e-08. After the first step, 529 NHP AD events and 2161 NSF AD events remained as potentially true domain events.

In the second step, we searched for alternative isoforms in the reference species (human/*S. cerevisiae*) in the Ensembl database, and checked whether an isoform was present that contained the AD. This step is only pertinent for the primate data set, since none of the *S. cerevisiae* genes corresponding to the potential AD events had known isoforms. For the NHP proteins, a total of 120 potential AD events were due to inconsistent isoform annotations.

**FIGURE 5:**
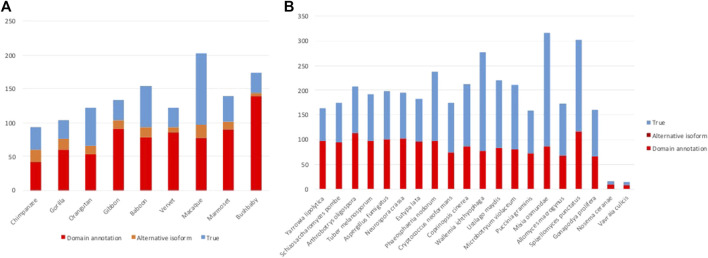
Results of error detection protocol for **(A)** NHP proteins and **(B)** NSF proteins with potential AD events.

After the different stages of error detection (Figure 5; Table 7, Table 8), only 397 NHP AD events out of 1,233 (32.2%) and 2163 NSF AD events out of 3785 (57.1%) are considered to be reliable candidates for true domain events. Of these, 0 NHP AD events and 126 (6.2%) NSF AD events were found in SwissProt proteins.

**TABLE 7:** Results of error detection protocol for NHP proteins with potential additional domain (AD) events. % true AD = % of potential AD that are identified as true AD after re-evaluation.

	Potential AD	Domain annotation	Alternative isoforms	True AD	% true AD
Chimpanzee	92	42	18	32	34.8
Gorilla	102	60	16	26	25.5
Orangutan	121	53	13	55	45.5
Gibbon	131	90	13	28	21.2
Baboon	154	79	15	60	39.0
Vervet	122	86	8	28	23.0
Macaque	199	77	20	102	51.3
Marmoset	139	90	12	37	26.6
Bushbaby	173	139	5	29	16.8
Total	1,233	716	120	397	32.2

**TABLE 8:** Results of error detection protocol for NSF proteins with potential additional domain (AD) events.

	Potential AD	Domain annotation	True AD	% true AD
*Yarrowia lipolytica*	163	97	66	40.5
*Schizosaccharomyces pombe*	174	94	80	46.0
*Arthrobotrys oligospora*	207	113	94	45.4
*Tuber melanosporum*	192	98	94	49.0
*Aspergillus fumigatus*	199	100	99	49.7
*Neurospora crassa*	195	102	93	47.7
*Eutypa lata*	183	96	87	47.5
*Phaeosphaeria nodorum*	237	98	139	58.6
*Cryptococcus neoformans*	174	72	104	59.1
*Coprinopsis cinerea*	212	87	125	59.0
*Wallemia ichthyophaga*	277	77	200	72.2
*Ustilago maydis*	221	83	138	62.4
*Microbotryum violaceum*	211	80	131	62.1
*Puccinia graminis*	159	73	86	54.1
*Mixia osmundae*	316	86	228	72.6
*Allomyces macrogynus*	173	67	106	61.3
*Spizellomyces punctatus*	302	116	186	61.6
*Gonapodya prolifera*	161	66	95	59.0
*Nosema ceranae*	15	9	6	40.0
*Vavraia culicis*	14	8	6	42.9
Total	3785	1,622	2,163	57.1

### 3.6 Origin of errors leading to false MD and AD events

Our evaluations of the potential MD and AD revealed that a significant proportion of the events can be attributed to systematic errors. Overall, inconsistent domain annotations accounted for the most errors (NHP: 42.7%, NSF: 46.7%). Gene prediction or isoform errors were the second most frequent causes of false positive domain events (NHP: 26.1%, NSF: 6.0%).

To further investigate the causes of the false domain events, we compared the rates of MD and AD events observed before and after the fact-checking protocol with two measures of genome and gene annotation quality. It should be noted that we excluded the two microsporidia species *N. ceranae* and *V. culicis* from the NSF dataset, due to the bias in the MD/AD ratio for these parasitic fungi. First, we used the contig N50 as an estimate of genome sequence/assembly completeness (from Table 1) and compared the N50 values to the frequency of domain events using graphical plots (Supplementary Figure S1) and Spearman rank correlations (Supplementary Table S1). For both NHP and NSF datasets, the correlation between N50 and the number of MD events is reduced after fact-checking (for NHP: potential MD R = −0.7, *p* = 0.04, true MD R = 0.2, *p* = 0.55; and for NSF: potential MD R = −0.4, *p* = 0.08, true MD R = −0.02, *p* = 0.93), suggesting that the fact-checking protocol was successful in removing this bias. However, the correlation between N50 and the number of AD events is more complex. For NHP, the correlation is increased for true AD events (R = 0.7, *p* = 0.037) compared to potential AD events (R = 0.2, *p* = 0.52). For NSF, no significant correlations were found for the AD events before or after fact-checking.

We then used the BUSCO complete metric (Manni et al., 2021) as a complementary measure to assess genome assembly and gene annotation completeness (from Table 1). BUSCO complete scores are based on the percentage of complete genes identified in a genome relative to a Benchmark of universal Single-Copy Orthologs. No significant correlations were observed between the BUSCO metric and the number of events before or after fact-checking (Supplementary Figure S1; Supplementary Table S1), although this may be due to the relatively small range of BUSCO values especially for NHP (BUSCO between 96.0 and 99.9).

We also compared the frequency of domain events before and after fact-checking to an estimate of the phylogenetic distance between the non-reference proteomes and the corresponding reference (human/*S. cerevisiae*). Intuitively, we would expect more true domain events in species that are more phylogenetically distant. However, it may also be true that identification of domain events in more distant species is more difficult, giving rise to more errors. We estimated the phylogenetic distance from publicly available genome-scale trees (see Methods) and performed a phylogenetic generalized least squares (PGLS) regression analysis to account for the phylogenetic relatedness of the species (Supplementary Table S1). For NHP, significant correlations were found for potential MD (*R* ^2^ = 0.61, *p* = 0.02) and true MD (*R* ^2^ = 0.71, *p* = 0.01), but not for potential AD (*R* ^2^ = 0.07, *p* = 0.5) or true AD (*R* ^2^ = 0.02, *p* = 0.7). For NSF, no significant correlations were observed, except for true MD (*R* ^2^ = 0.23, *p* = 0.04). Thus, our fact-checking protocol generally increases the correlation between phylogenetic distance and the number of predicted events, at least for MD, supporting the hypothesis that true MD events occur more frequently in more distantly related species. Again, the correlation for the number of AD events is more complex, and potential reasons for this are discussed below.

The proportion of potential MD events identified as true events is different for the two datasets: 9.2% for the NHP proteomes, and 31.2% for the NSF proteomes. The proportion of true AD events is also different: 32.2% for the NHP proteomes, 57.1% for the NSF proteomes. The larger proportions of true AD compared to true MD for both NHP and NSF are possibly due to protocol issues since false positive MD can be more easily identified by checking for their presence in the corresponding genome sequence. It is more difficult to affirm that potential AD are in fact false positives, for example resulting from erroneous gene fusions or alternative start/stop codons. Similar issues have been observed previously at the gene level, where it is generally accepted that in eukaryotic genome evolution gene loss is prevalent over gene gain (Deutekom et al., 2019). However, due to sequencing issues or incorrect gene prediction, genes can be falsely inferred as absent, implying that loss estimates may be overestimated.

### 3.7 Impact of errors on estimates of MD and AD events

**FIGURE 6:**
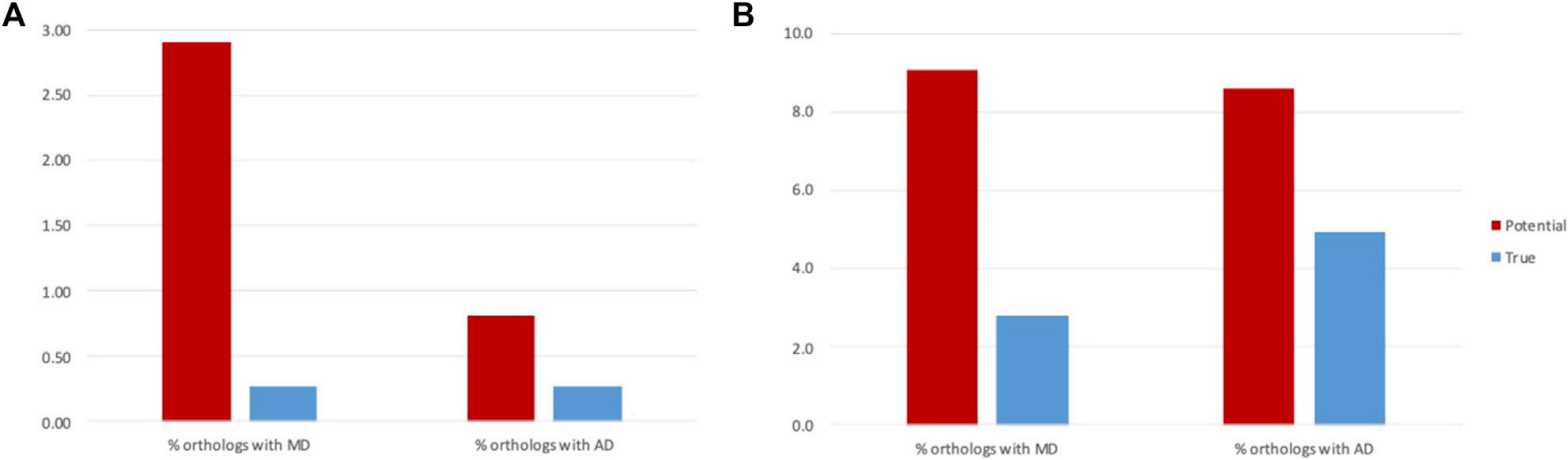
Rates of MD and AD events before and after filtering of false positives for **(A)** NHP and **(B)** NSF proteins.

The error detection protocol presented in the previous section allowed to filter false positive predictions and to more reliably estimate the rates of MD and AD events in the NHP/NSF proteomes (Figure 6). After filtering, the mean number of NHP orthologs with different domain contents is reduced from 3.7% to 0.5%. This is in line with previous observations (Nagy et al., 2011), for example for human-orangutan comparisons, a rate of 0.3% orthologs with different domain architectures was identified in expert-reviewed proteins from the SwissProt database. Filtering of the NSF dataset also resulted in a reduction of the number of orthologs with different domain contents from 17.6% to 7.7%, although the proportion of true events is larger than for NHP proteins.

For the NHP dataset, the potential MD events inferred from the public database annotations are more frequent than AD events (2.9% of orthologs with MD and 0.8% of orthologs with AD: paired *t*-test, t (10) = -5.70, *p* = 0.0005). However, we conclude that this difference is due to annotation errors, since after filtering of the false positives, MD and AD rates are the same (0.27% of orthologs with MD and 0.26% of orthologs with AD: paired *t*-test t (10) = -0.10, *p* = 0.92). Similar rates of MD and AD might be expected, since the absence/presence of a domain was arbitrarily defined to refer to the NHP protein compared to the human reference.

In contrast, for the NSF dataset, while the number of potential MD and AD events inferred from the public database annotations are similar (9.1% of orthologs with MD and 8.6% of orthologs with AD: paired *t*-test, t = −1.01, *p* = 0.33), filtering of false positives led to more frequent AD in NSF with respect to *S. cerevisiae* than MD (2.8% of orthologs with MD and 4.9% of orthologs with AD: paired *t*-test, t (20) = 2.61, *p* = 0.02). One reason for the higher rate of AD compared to MD may be the higher diversity of fungal genomes due to their diverse lifestyles and specific adaptations to natural or laboratory ecosystems (Naranjo-Ortiz and Gabaldón, 2019).

**FIGURE 7:**
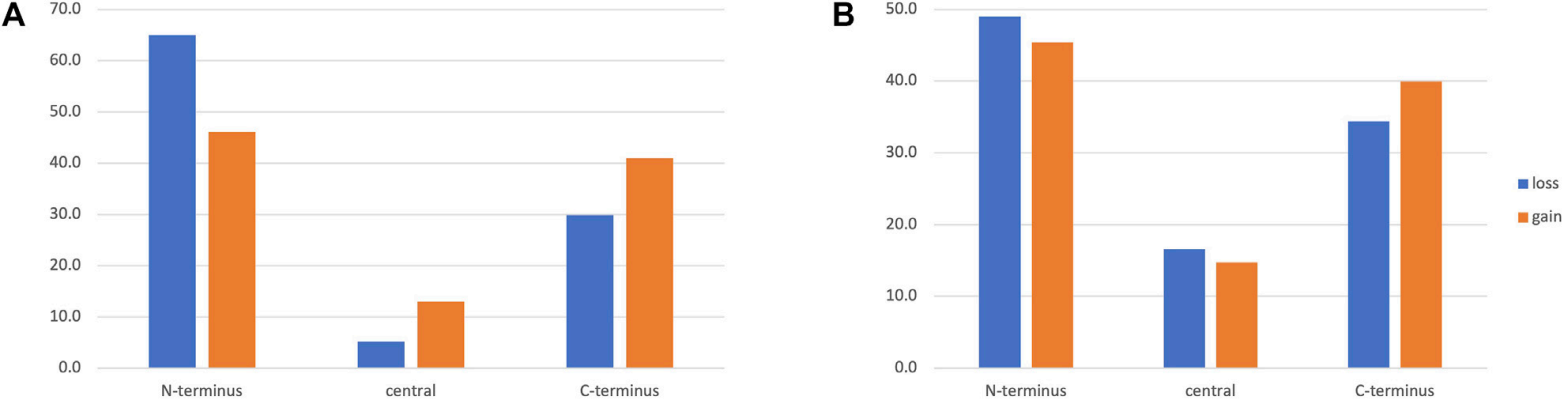
Location of MD and AD events at the N/C-termini or in the middle of the protein for **(A)** NHP and **(B)** NSF proteins.

Finally, we investigated the position of the true MD and AD events within the protein sequence. For domain events where the reference protein and the ortholog had at least two annotated domains, we determined whether the MD or AD was found at the N/C-termini or in the middle of the protein. For both NHP and NSF datasets (Figure 7), the MD and AD events were less frequent in the middle of the protein and N-terminal events were slightly more frequent than C-terminal events, in line with previous findings (Dohmen et al., 2020).

### 3.8 Characterization of true domain events in primates

In this section, we perform an initial evolutionary and functional investigation of the 808 domain events (411 MD and 397 AD) that were retained as true events in the primate dataset. Our initial error detection protocol identified AD and MD at the level of Pfam protein families. However, many Pfam families are related and can be classified hierarchically into clans. Pfam defines a clan as a collection of families that have arisen from a single evolutionary origin. Although domain families in the same clan are considered to be homologous, they are so evolutionarily diverse that the family members cannot be identified with a single HMM. Of the 808 domain events, 52 events (44 MD, 8 AD) involved two domains in the same Pfam clan and were excluded from the subsequent analyses. The distribution of the remaining 756 events is provided in Supplementary Table S2.

It has been proposed that novel combinations of preexisting domains had a major role in the evolution of protein networks and more complex cellular activities (Peisajovich et al., 2010). In agreement with this, we found that the most frequent Pfam-A domains implicated in MD or AD events in the primate dataset (Supplementary Table S3) are mainly involved in signaling or regulatory functions, including (i) the Kruppel associated box (KRAB) domain and zinc finger domains (PF14634, PF00096, PF02023) found in diverse transcription factors, (ii) the Ankyrin repeat and SAM domain commonly involved in protein-protein interactions, and (iii) the Src homology-3 frequently having a role in signaling pathways. Other domains are present in immune system proteins (PF13895, PF07679), or cytoskeletal-associated proteins (PF12796, PF00307, PF00373, PF15974), or are found in proteins involved in diverse processes (PF14604, PF01462, PF13202, PF00100).

**FIGURE 8:**
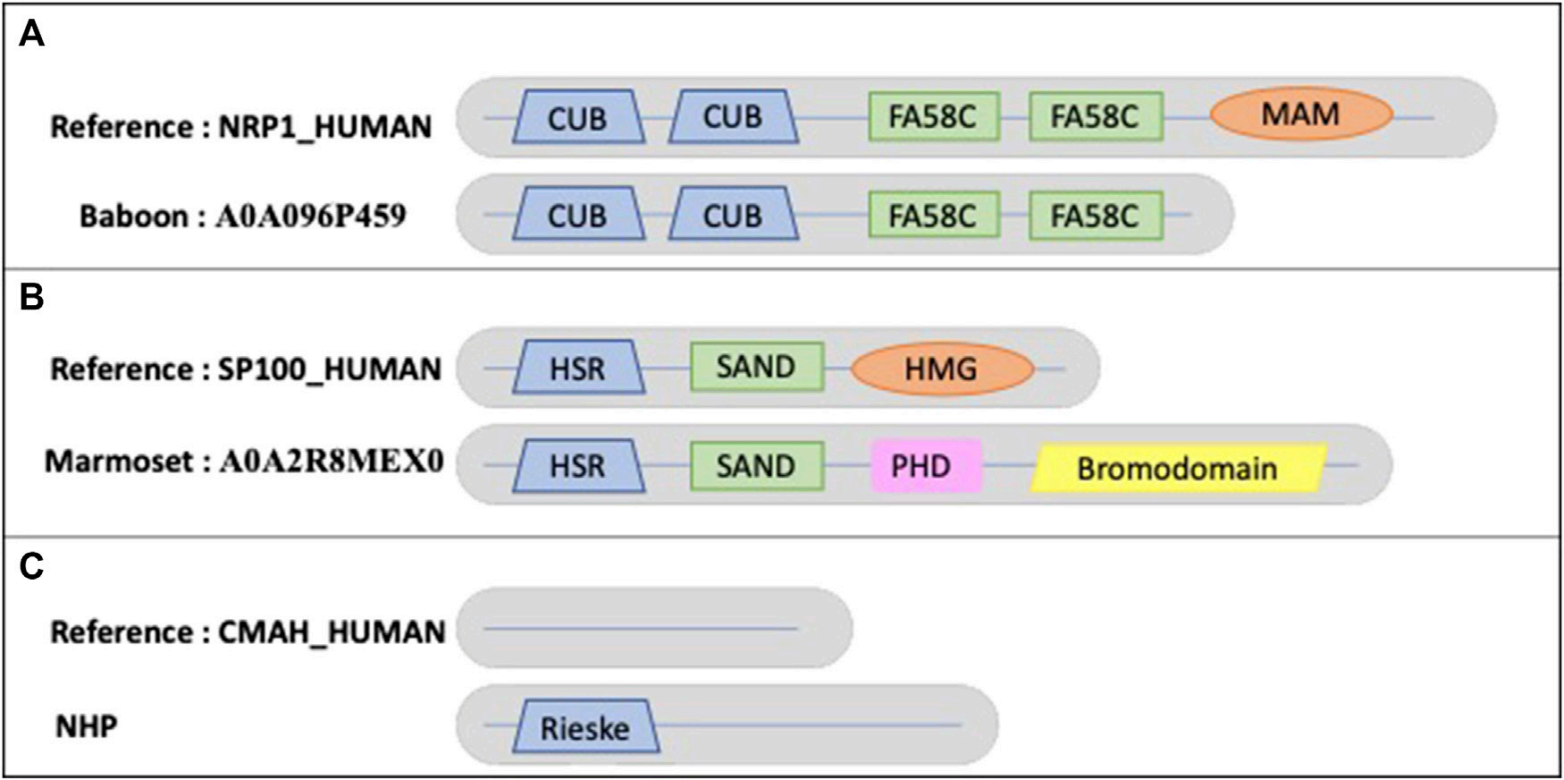
Schematic views of the example domain events observed in NHP proteomes. **(A)**. Domain organizations for human protein NRP1_HUMAN and baboon protein A0A096P459, showing the shared domains CUB (PF00431) and FA58C (PF00754) and the missing MAM (PF00629) domain in baboon. **(B)**. Domain organizations for human protein SP100_HUMAN and marmoset protein A0A2R8MEX0, showing the shared domains HSR (PF03172) and SAND (PF01342) and the missing HMG (PF00505) domain in marmoset. The marmoset protein contains 2 additional domains: PHD (PF00628) and bromodomain (PF00439). **(C)**. Domain organizations for human protein CMAH_HUMAN and all NHP orthologs, showing the additional Rieske-type domain (PF00355) in NHP orthologs.

Figure 8A shows an example of a true MD in an NHP protein, where the baboon ortholog (A0A096P459) has a missing MAM domain (PF00629) compared to the human sequence NRP1_HUMAN (O14786). NRP1_HUMAN is defined as neuropilin-1, a vascular endothelial cell growth factor receptor that plays roles in angiogenesis, axon guidance, cell survival, migration, and invasion. It also recognizes and binds to a specific motif on SARS-CoV-2 spike protein S1 and enhances SARS-CoV-2 infection. The human MAM domain consists of approximately 170 amino acids and is likely to have an adhesive function. Truncation of the MAM domain abolishes the ability of neuropilin to mediate semaphorin-induced neuronal growth collapse (Lu et al., 2021).

Another example (Figure 8B) is the New World marmoset ortholog (A0A2R8MEX0) with a missing domain (PF09011) compared to the human sequence SP100_HUMAN (P23497). The nuclear autoantigen Sp-100 plays a role in angiogenesis, controlling endothelial cell motility and invasion. It also plays a role in infection by viruses, including human cytomegalovirus and Epstein-Barr virus. In Old World monkeys, including apes and humans, one of the splicing variants is extended by an HMG1L3 retrotransposition into the SP100 locus that occurred after divergence of New World and Old World monkey lineages (Devor, 2001). This retrotranscribed copy was inserted at the 3′end of the SP100 gene and has become incorporated into the 3′end of the SP100 locus as an exon, resulting in the addition of a DNA-binding function to the SP100 protein.

An example of a true AD event is observed in all NHP orthologs of the human protein CMAH_HUMAN (Q9Y471), an inactive cytidine monophosphate-N-acetylneuraminic acid (CMP-Neu5Gc) hydroxylase (Figure 8C). The human protein is N-terminally truncated compared to orthologs and lacks a region containing the Rieske-type iron-sulfur cluster domain (PF00355). It has been suggested that the inactive protein leads to the fact that humans differ from other primates because they completely lack Neu5Gc on their cell surfaces (Suntsova, Buzdin, 2020).

### 3.9 Characterization of true domain events in fungi

In this section, we investigate the 3406 domain events (1243 MD and 2163 AD) that were retained as true events in the fungi dataset. As for primates, we first checked for MD/AD in the same Pfam clan. Of the 3406 domain events, 567 (240 MD, 314 AD) involved two domains in the same Pfam clan and were excluded from the subsequent analyses. The distribution of the remaining 2,852 events is provided in Supplementary Table S4.

Supplementary Table S5 shows the top 10 most frequent Pfam-A domains implicated in MD or AD in the fungi dataset. Several domains are also present in the primate top 10 list, including the promiscuous domains (C2H2, RING, EF hand, Ankyrin repeats) found in diverse proteins. Other promiscuous domains include the WD, RWD, F-box-like domains. For some NSF MD, the domain is missing in all orthologs and thus most likely correspond to a domain gain in the reference protein rather than independent domain loss events in multiple NSF. These domains include homing endonucleases (PF05203, PF05204) encoded by mobile DNA elements, the N-terminal domain of methionyl-tRNA synthetase (MetRS), or the C-terminal domain of the mitochondrial ribosomal L27 protein.

**FIGURE 9:**
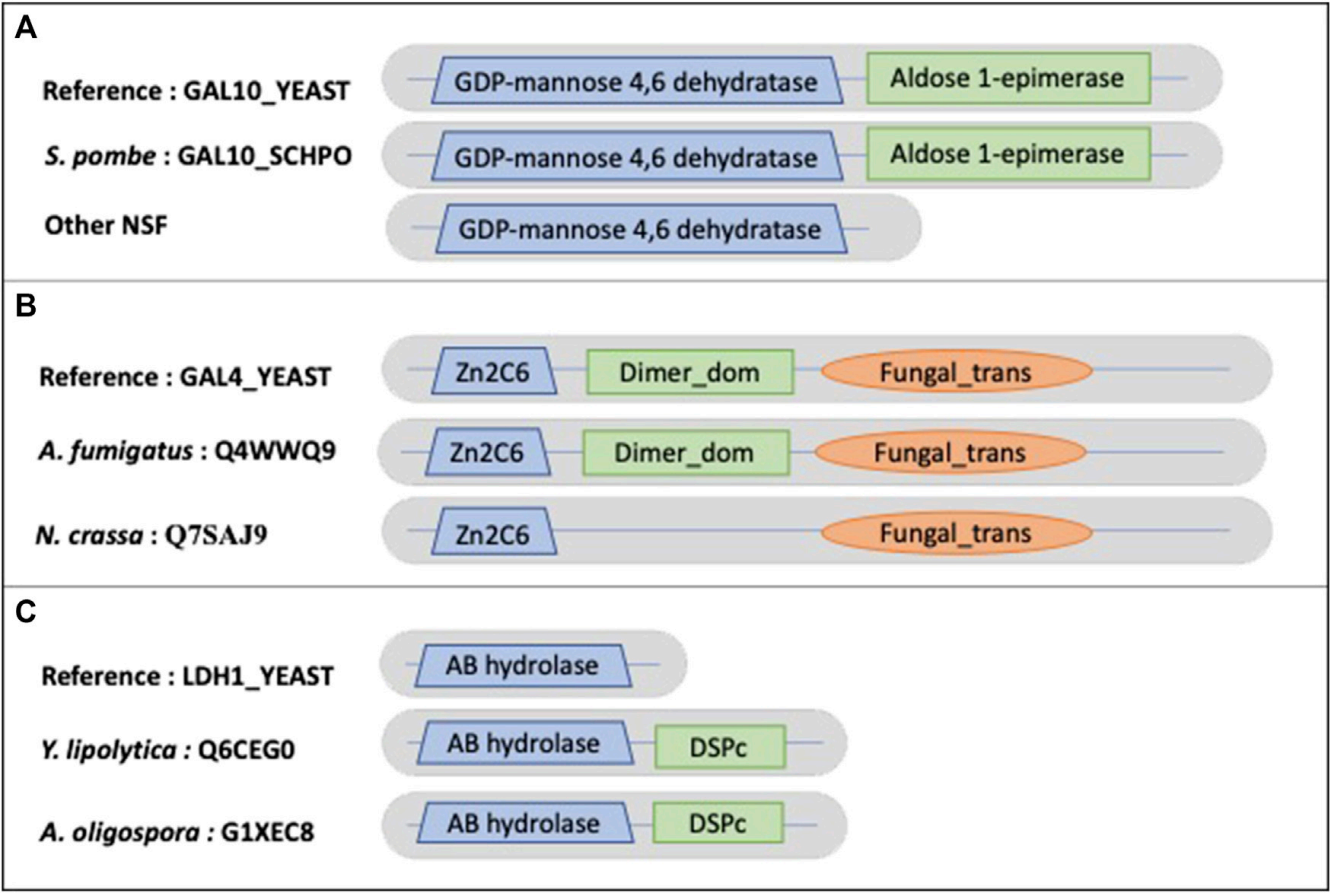
Schematic views of the example domain events observed in NSF proteomes. **(A)**. Domain organizations for *Saccharomyces cerevisiae* protein GAL10_YEAST, *Schizosaccharomyces pombe* protein GAL10_SCHPO and other NSF orthologs, showing the shared domain GDP-mannose 4,6 dehydratase (PF16363) and the missing Aldose 1-epimerase (PF01263) domain in NSF orthologs. **(B)**. Domain organizations for *Saccharomyces cerevisiae* protein GAL4_YEAST, *A. fumigatus* protein Q4WWQ9 and *Neurospora crassa* protein Q7SAJ9, showing the shared Zn2C6 (PF00172) and Fungal_trans domains (PF04082) and the missing dimerization domain (Dimer_dom) (PF03902) in the *Neurospora crassa* protein. **(C)**. **(B)**. Domain organizations for *Saccharomyces cerevisiae* protein LDH1_YEAST, *Y. lipolytica* protein Q6CEG0 and *A. oligospora* protein G1XEC8, showing the shared AB hydrolase (PF12697) domain and the additional DSPc (PF00782) domain in *Y. lipolytica* and *A. oligospora* proteins.

Other examples of true NSF MD events include the galactose metabolic process*.* Most hemiascomycetes can grow on galactose as a sole carbon source, and in *S. cerevisiae* seven genes (the GAL genes) function exclusively in this pathway. Although orthologs of the GAL genes are present in many yeast genomes, they have been lost independently in several lineages (Hittinger et al., 2004). This specialty is also reflected at the protein domain level. The example shown in Figure 9A concerns GAL10_YEAST (P04397), a bifunctional protein containing a GDP-mannose 4,6 dehydratase domain (PF16363) and an Aldose 1-epimerase domain (PF01263). The 17 identified NSF orthologs (no orthologs were found for *Gonapodya prolifera, Nomascus cernae, V. culicis*) all share the GDP-mannose 4,6 dehydratase domain. However, only *Schizosaccharomyces pombe* exhibits both domains, while 4 orthologs correspond to partially uncharacterized genome regions and 12 have confirmed losses of the Aldose 1-epimerase domain. Figure 9B shows another GAL protein, GAL4_YEAST (P04386), a positive regulator for the gene expression of the galactose-induced genes that code for enzymes used to convert galactose to glucose. *Cryptococcus neoformans* is missing the DNA binding domain (PF00172), but the corresponding genome sequence is partially uncharacterized. *A. oligospora* has the dimerization domain (PF03902) after correction of the gene prediction. The loss of the dimerization domain is confirmed in the other identified orthologs.

An example of a NSF AD is observed in the *Y. lipolytica* ortholog of LDH1_YEAST (P38139), lipid droplet hydrolase 1, that plays a role in maintaining lipid homeostasis in *S. cerevisiae* (Figure 9C). *Yarrowia lipolytica* is a model microorganism for lipid production because of its ability to accumulate high levels of lipids. LDH1_YEAST shares an alpha/beta hydrolase fold domain (PF00561) with *Y. lipolytica* and *A. oligospora*, but these two orthologs also contain a C-terminal dual specificity phosphatase, catalytic domain (PF00782).

More complex MD/AD events are found in orthologs of the reference protein FOX2_YEAST (Q02207), a peroxisomal hydratase-dehydrogenase-epimerase trifunctional protein, with 17 identified NSF orthologs (no orthologs were found for *S. pombe* and the two Microsporidia). The MaoC like domain (PF01575), with possible hydratase activity, is lost in the two Chytridiomycota, and the Basidiomycota *W. ichthyophaga* and *Coprinopsis cinerea*. In *C. cinerea*, the MaoC like domain is found in a separate downstream gene, suggesting a possible fusion/fission event.

## 4 Discussion

Analysis of domain architecture changes is important for fundamental studies of evolution and applications in the use of non-human primates in medical studies, for example (Schmidt et al., 2022), or in the search of new antifungal targets (Barrera et al., 2014). Here, we have shown that on average domain losses are over-estimated ten-fold and three-fold in the NHP and NSF proteins respectively, in line with previous studies (Nagy et al., 2011). For the first time, we also show that domain gains are over-estimated by three-fold and two-fold respectively in NHP and NSF proteins. The proportion of potential NHP events that are identified as true remains low, even for the most reliable proteomes (26% true events for macaque, and 60% for *Ustilago maydis*)*,* clearly demonstrating the necessity for reliable quality control and error detection.

After error filtering, true domain events are observed with a frequency of 0.5% for NHP orthologs and 7.7% for NSF orthologs. This difference highlights the different nature of the two datasets used in this work. The datasets were chosen to represent the main challenges for domain annotation pipelines. The first dataset includes closely related NHP species (sharing >89% protein sequence identity with human) with a common ancestor that appeared recently around 50–55 Mya (Martin, 2012). True domain events are thus expected to occur at relatively low rates. Nevertheless, the annotation of NHP genomes poses significant challenges due to their large intron-rich and repeat-rich genomes and the variable genome sequence quality (Wu D. D. et al., 2022) implying that errors are still common (Meyer et al., 2020). In view of the clear physiological, cognitive and behavioral differences between NHP species, high quality data is crucial for comparative studies aimed at describing to what extent genetic differences drive phenotypic differences (Rogers and Gibbs, 2014). In contrast, the NSF species of the second dataset generally have smaller genomes of high quality and few introns meaning that genes are theoretically easier to predict. However, the NSF have evolved over a longer time period (the fungal stem lineage emerged 1,000 Mya) and adapted to very diverse environments and lifestyles (Berbee et al., 2017). Again, error-free data are required to accurately identify different adaptive evolutionary trajectories.

The fact-checking method used here confirms that errors or inconsistencies are widespread in the protein database entries corresponding to the two datasets. For both NHP and NSF, the most frequent source of errors was heterogeneous domain annotations between orthologs. InterPro combines predictive models from several different databases, including Pfam into a single resource. The entire InterPro database is regularly reviewed for accuracy, and matches for sequences from UniprotKB are updated or removed where necessary (Blum et al., 2021). These efforts clearly improve the accuracy and coverage of protein domain databases, especially for the well-studied model species. Unfortunately, it also implies that domain annotations can be inconsistent between related organisms, thus hindering systematic large-scale comparative studies.

Gene prediction failures were the second most frequent source of errors, particularly for NHP proteins. The relative difference between NHP and NSF is probably explained by the fact that the problems of gene prediction are less severe in the case of fungi that generally have less intron-rich genomes than primates. Furthermore, in primates, some potential domain events also reflect the presence of alternative splicing isoforms, and the fact that the UniprotKB database may define different canonical isoforms for the orthologs of different species.

Finally, 22% of domain events for NHP and 7% for NSF, were mapped to incomplete or uncharacterized genome sequences. In this case, the protocol could not distinguish between true and false events. Nevertheless, genome sequence quality should improve in the near future with the application of long read sequencing technologies, for example.

Three important caveats should be kept in mind. First, we extracted ortholog information from the OrthoInspector database, which identifies pairwise orthologs based on protein sequence comparisons. This implies that orthologs with very different domain architectures may be missed by the algorithm, and thus our list of domain events may be incomplete. In contrast, we assumed that the identified ortholog pairs are accurate, since OrthoInspector achieved a precision >99% in independent benchmarking tests (Altenhoff et al., 2020). Although the OrthoInspector database includes one-to-many and many-to-many orthologs, we restricted our analyses to orthologs with one-to-one relationships, thus excluding complex gene families that may have undergone copy number variations and subsequent sequence divergence through neo- or sub-functionalization.

Second, our more relaxed definition of the ‘domain content’ of proteins considers only the set of domains present in a protein irrespective of domain order or domain duplications. This definition focuses on the important evolutionary events involving loss or gain of unique domains and has been used previously to study evolution of multidomain proteins (Przytycka et al., 2006; Moore and Bornberg-Bauer, 2012). Nevertheless, more stringent definitions of domain architecture have been proposed, such as the linear sequence of constituent domains from the N-terminus to the C-terminus (Nagy et al., 2011; Dohmen et al., 2020), which would allow to address more complex domain-level events.

Third, it should be noted that our protocol uses pairwise sequence comparisons and MD/AD events are defined with respect to the reference protein (human or *S. cerevisiae*). This does not necessarily reflect the underlying evolutionary mechanisms and cannot distinguish between a domain being recruited in one protein or lost in the other. Therefore, our study does not provide further evidence concerning the debate about the relative rates of domain losses and gains in a given clade. Resolution of the evolutionary mechanisms underlying the domain content differences observed in this work would require detailed phylogenetic analyses and reconstruction of ancestral states.

Based on the true events, we performed some preliminary analyses to investigate the functional relevance of our findings. After filtering of errors, similar rates of MD and AD are observed in the closely related NHP, while AD are more frequent than MD in the NSF that have adapted to more specific ecosystems. The latter is in line with previous findings that emergence of novel domains is foremost associated with environmental adaptations (Moore and Bornberg-Bauer, 2012). The most frequently lost/gained domains often reflect known species specificities, notably immune system proteins or zinc-finger transcription factors in primates (Rogers and Gibbs, 2014), or key metabolic processes in fungi (Wu B. et al., 2022). We also confirmed that domain events are predominantly observed on sequence termini, and more frequently on the N-terminus, than in the internal part of the architecture. It has been hypothesized that insertions of new transcription start and stop codons, as well as gene fusion and fission, are more likely to occur than, for example, intron mobility caused by exon shuffling (Buljan and Bateman, 2009).

In the future, we plan to extend the functional analyses to investigate the potential enrichment of the domain gain/loss events observed here in specific cellular pathways or processes. We will also apply the fact-checking approach to other domain events, such as domain shuffling or domain duplications, observed in primate and fungi proteins. An important application will be the comparison of the evolution of domain architectures between orthologs and paralogs. For example, are domain architectures more similar between orthologs than paralogs at the same degree of evolutionary separation? While orthologs are generally expected to experience stronger evolutionary pressure to maintain the same function than paralogs, it would be interesting to test whether functional conservation is associated with a higher conservation of domain architectures, i.e., if domain architecture is an important vehicle of protein function.

## Data Availability

The datasets presented in this study can be found in online repositories. The names of the repository/repositories and accession number(s) can be found in the article/Supplementary Material.
