# Supplementary material for: Real or fake? Measuring the impact of protein annotation errors on estimates of domain gain and loss events
Source: Front Bioinform. 2023 Apr 20;3:1178926. doi: 10.3389/fbinf.2023.1178926 (PMC10158824; doi:10.3389/fbinf.2023.1178926)
Supplement: Supplementary file 2 [file Table3.DOCX]

Supplementary Material

Real or fake? Measuring the impact of protein annotation errors on estimates of domain gain and loss events

Arnaud Kress, Olivier Poch, Odile Lecompte, Julie D. Thompson*

*** Correspondance:** Julie D. Thompson: [thompson@unistra.fr](mailto:thompson@unistra.fr)

# Supplementary Figures

Figure S1. Scatter plots of the frequency of potential and true domain events (calculated as the number of domain events divided by the number of orthologs) for NHP and NSF species with genome quality (contig N50 and BUSCO complete) metrics. Trend lines for each dataset are shown as dotted lines. The two microsporidia species *N. ceranae* and *V. culicis* were excluded due to a bias in the MD/AD ratio.

# Supplementary Tables

**Table S1.** Correlation of the frequency of potential and true domain events (calculated as the number of domain events divided by the number of orthologs) with genome quality (contig N50 and BUSCO complete) and phylogenetic distance metrics, for A. NHP and B. NSF species, excluding the two microsporidia species *N. ceranae* and *V. culicis* due to a bias in the MD/AD ratio. Significant correlations (p<0.05) are highlighted in bold.

1. NHP

|  | Spearman rank correlation | | PGLS |
| --- | --- | --- | --- |
|  | *Contig N50* | *BUSCO complete* | *Phylogenetic distance* |
| Potential MD | **R=-0.7, p=0.04** | R=0.1, p=0.74 | **R^2^=0.61, p=0.02** |
| True MD | R=0.2, p=0.55 | R=0.1, p=0.84 | **R^2^=0.71, p=0.01** |
|  | | | |
| Potential AD | R=0.2, p=0.52 | R=0.3, p=0.46 | R^2^=0.07, p=0.54 |
| True AD | **R=0.7, p=0.04** | R=0.2, p=0.61 | R^2^=0.02, p=0.74 |

1. NSF

|  | *Contig N50* | *BUSCO complete* | *Phylogenetic distance* |
| --- | --- | --- | --- |
| Potential MD | R=-0.4, p=0.08 | R=-0.3, p=0.27 | R^2^=0.05, p=0.37 |
| True MD | R=-0.02, p=0.93 | R=-0.8, p=0.47 | R^2^=**0.23, p=0.04** |
|  | | | |
| Potential AD | R=0.03, p=0.91 | R=-0.1, p=0.69 | R^2^=0.05, p=0.39 |
| True AD | R=-0.07, p=0.79 | R=-0.1, p=0.59 | R^2^=0.07, p=0.29 |

**Table S2. True NHP domain events (excluding those involving two domains in the same Pfam clan).**

|  | True MD | True AD | Ratio  MD/AD | Total |
| --- | --- | --- | --- | --- |
| Chimpanzee | 8 | 32 | 0.25 | 40 |
| Gorilla | 36 | 26 | 1.38 | 62 |
| Orangutan | 34 | 55 | 0.62 | 89 |
| Gibbon | 17 | 26 | 0.65 | 43 |
| Baboon | 54 | 58 | 0.93 | 112 |
| Vervet | 109 | 26 | 4.19 | 135 |
| Macaque | 49 | 100 | 0.49 | 149 |
| Marmoset | 27 | 37 | 0.73 | 64 |
| Bushbaby | 33 | 29 | 1.14 | 62 |
| Total | 367 | 389 | 0.94 | 756 |

**Table S3A. Top 10 most frequent Pfam domains observed in MD events in NHP proteins.**

| Pfam identifier | Total no. of human proteins with domain | Domain events | | Description |
| --- | --- | --- | --- | --- |
|  |  | No. of human proteins | No. of NHP proteins |  |
| PF01352 | 117 | 17 | 17 | Kruppel associated box |
| PF14634 | 66 | 9 | 10 | Zinc-RING finger domain |
| PF14604 | 40 | 7 | 8 | Variant SH3 domain |
| PF13895 | 491 | 7 | 7 | Immunoglobulin domain |
| PF12796 | 919 | 5 | 6 | Ankyrin repeats |
| PF00307 | 356 | 4 | 5 | Calponin homology (CH) domain |
| PF00096 | 2004 | 4 | 5 | Zinc finger, C2H2 type |
| PF00069 | 1786 | 4 | 4 | Protein kinase domain |
| PF01462 | 120 | 4 | 4 | Leucine rich repeat N-terminal domain |
| PF02023 | 240 | 4 | 4 | SCAN domain |
| PF07679 | 591 | 4 | 4 | Immunoglobulin I-set domain |
| PF15974 | 53 | 4 | 4 | Cadherin C-terminal cytoplasmic tail, catenin-binding region |

**Table S3B. Top 10 most frequent Pfam domains observed in AD events in NHP proteins.**

| PF01352 | 1053 | 10 | 17 | Kruppel associated box |
| --- | --- | --- | --- | --- |
| PF02809 | 187 | 2 | 10 | Ubiquitin Interacting Motif |
| PF00096 | 2004 | 2 | 9 | Zinc finger, C2H2 type |
| PF13202 | 94 | 4 | 8 | EF hand |
| PF00373 | 226 | 2 | 8 | FERM central domain |
| PF12012 | 14 | 1 | 7 | Domain of unknown function 3504 |
| PF13873 | 19 | 1 | 6 | Myb/SANT-like DNA-binding domain |
| PF01249 | 10 | 1 | 6 | Ribosomal protein S21e |
| PF00130 | 246 | 4 | 6 | Phorbol esters/diacylglycerol binding domain |
| PF00100 | 52 | 2 | 6 | Zona pellucida-like domain |

**Table S4. True NSF domain events (excluding those involving two domains in the same Pfam clan).**

|  | True MD | True AD | Ratio  MD/AD | Total |
| --- | --- | --- | --- | --- |
| *Yarrowia lipolytica* | 44 | 50 | 0.88 | 94 |
| *Schizosaccharomyces pombe* | 46 | 61 | 0.75 | 107 |
| *Arthrobotrys oligospora* | 39 | 78 | 0.50 | 117 |
| *Tuber melanosporum* | 44 | 77 | 0.57 | 121 |
| *Aspergillus fumigatus* | 34 | 81 | 0.42 | 115 |
| *Neurospora crassa* | 41 | 77 | 0.53 | 118 |
| *Eutypa lata* | 35 | 69 | 0.51 | 104 |
| *Phaeosphaeria nodorum* | 53 | 129 | 0.41 | 182 |
| *Cryptococcus neoformans* | 59 | 90 | 0.66 | 149 |
| *Coprinopsis cinerea* | 58 | 105 | 0.55 | 163 |
| *Wallemia ichthyophaga* | 66 | 186 | 0.35 | 252 |
| *Ustilago maydis* | 66 | 117 | 0.56 | 183 |
| *Microbotryum violaceum* | 50 | 115 | 0.43 | 165 |
| *Puccinia graminis* | 48 | 72 | 0.67 | 120 |
| *Mixia osmundae* | 59 | 213 | 0.28 | 272 |
| *Allomyces macrogynus* | 40 | 91 | 0.44 | 131 |
| *Spizellomyces punctatus* | 46 | 150 | 0.31 | 196 |
| *Gonapodya prolifera* | 63 | 77 | 0.82 | 140 |
| *Nosema ceranae* | 40 | 6 | 6.67 | 46 |
| *Vavraia culicis* | 72 | 5 | 14.40 | 77 |
| Total | 1003 | 1849 | 0.54 | 2852 |

**Table S5A. Top 10 most frequent Pfam domains observed in MD events in NSF.**

| Pfam identifier | Total no. of *S. cerevisiae* proteins with domain | 1. Domain events | | Description |
| --- | --- | --- | --- | --- |
|  |  | No. of *S. cerevisiae* proteins | 1. No. of NSF proteins |  |
| 1. PF05204 | 1. 2 | 1. 1 | 1. 18 | 1. Homing endonuclease |
| PF05203 | 2 | 1. 1 | 1. 18 | 1. Hom_end-associated Hint |
| PF00400 | 87 | 1. 7 | 1. 18 | 1. WD domain, G-beta repeat |
| PF09635 | 1 | 1. 1 | 1. 17 | 1. MetRS-N binding domain |
| PF00096 | 23 | 3 | 1. 17 | Zinc finger, C2H2 type |
| PF14634 | 1 | 1. 4 | 1. 16 | 1. zinc-RING finger domain |
| PF12209 | 1 | 1 | 1. 16 | Leucine permease transcriptional regulator helical domain |
| PF18471 | 1 | 1. 1 | 1. 15 | 1. Ribosomal L27 protein C-terminal domain |
| PF11549 | 1 | 1. 1 | 1. 15 | 1. Protein transport protein SEC31 |
| PF03725 | 3 | 1. 2 | 1. 15 | 1. 3' exoribonuclease family, domain 2 |

**Table S5B. Top 10 most frequent Pfam domains observed in AD events in NSF.**

| PF00400 | 87 | 18 | 41 | WD domain, G-beta repeat |
| --- | --- | --- | --- | --- |
| PF00098 | 4 | 7 | 29 | Zinc knuckle |
| PF12796 | 10 | 10 | 25 | Ankyrin repeats |
| PF00439 | 10 | 4 | 23 | Bromodomain |
| PF13202 | 2 | 3 | 19 | EF hand |
| PF04082 | 26 | 8 | 16 | Fungal specific transcription factor domain |
| PF05773 | 4 | 2 | 15 | RWD domain |
| PF00096 | 23 | 6 | 15 | Zinc finger, C2H2 type |
| PF12937 | 5 | 8 | 14 | F-box-like |
| PF01424 | 2 | 1 | 14 | R3H domain |
